# Mycotoxin Analysis of Human Urine by LC-MS/MS: A Comparative Extraction Study

**DOI:** 10.3390/toxins9100330

**Published:** 2017-10-19

**Authors:** Laura Escrivá, Lara Manyes, Guillermina Font, Houda Berrada

**Affiliations:** Laboratory of Food Chemistry and Toxicology, Faculty of Pharmacy, University of Valencia, Av. Vicent Andres Estelles s/n, 46100 Burjassot, Spain; laura.escriva@uv.es (L.E.);lara.manyes@uv.es (L.M.); guillermina.font@uv.es (G.F.)

**Keywords:** mycotoxins, urine, optimization, method validation, LC-MS/MS

## Abstract

The lower mycotoxin levels detected in urine make the development of sensitive and accurate analytical methods essential. Three extraction methods, namely salting-out liquid–liquid extraction (SALLE), miniQuEChERS (quick, easy, cheap, effective, rugged, and safe), and dispersive liquid–liquid microextraction (DLLME), were evaluated and compared based on analytical parameters for the quantitative LC-MS/MS measurement of 11 mycotoxins (AFB1, AFB2, AFG1, AFG2, OTA, ZEA, BEA, EN A, EN B, EN A1 and EN B1) in human urine. DLLME was selected as the most appropriate methodology, as it produced better validation results for recovery (79–113%), reproducibility (RSDs < 12%), and repeatability (RSDs < 15%) than miniQuEChERS (71–109%, RSDs <14% and <24%, respectively) and SALLE (70–108%, RSDs < 14% and < 24%, respectively). Moreover, the lowest detection (LODS) and quantitation limits (LOQS) were achieved with DLLME (LODs: 0.005–2 μg L^−1^, LOQs: 0.1–4 μg L^−1^). DLLME methodology was used for the analysis of 10 real urine samples from healthy volunteers showing the presence of ENs B, B1 and A1 at low concentrations.

## 1. Introduction

Toxic fungal secondary metabolites, known as mycotoxins, frequently contaminating food and feed are of concern due to their association with a wide array of adverse health effects [1,2]. The diversity of mycotoxins leads to a broad range of acute and chronic toxic effects in animals and humans, such as vomiting, hematotoxicity, immunosuppression, hepatotoxicity, nephrotoxicity, teratotoxicity, immunotoxicity, and hormonal or reproductive effects, although potencies vary depending on species and sex [3]. Humans are often simultaneously exposed to mycotoxins mixtures along with other contaminants such as pesticides or heavy metals, making multi-mycotoxin exposure study relevant from a public health perspective. Actual exposure to mycotoxins is difficult to measure using an indirect approach based on mycotoxin occurrence in food combined with data on food consumption. Individual exposure is influenced by the heterogeneous distribution of mycotoxins, under- and overestimation of food consumption data, the presence of masked mycotoxins, and individual differences in absorption, distribution, metabolism and excretion (ADME) [4]. To overcome these disadvantages, detecting the presence of mycotoxins in biological fluids such as blood and urine could be useful and reliable in short- and long-term exposure assessment, and may make it possible to predict future adverse health consequences [2]. Urine is the body fluid most often used to measure mycotoxin exposure due to large amounts being easily and non-invasively collected, although blood (serum, plasma) has also been used [4]. Hence, detection of mycotoxins in human or animal urine allows more accurate and objective exposure assessment at an individual level since it covers exposure from all sources, thus reducing uncertainties related to occurrence and consumption rates [5]. On the other hand, urine analysis may provide widely valuable information, from mycotoxins toxicokinetics, ADME and bioavaliability studies, to human biomonitoring and exposure assessment. Urine analysis of mycotoxins can be used to establish population reference ranges and identify vulnerable consumer groups and individuals with higher exposures [6]. Moreover, the relationship between urinary mycotoxin levels and some diseases such as nephropathy has been also investigated [7].

Since the advent of the latest generation of high-performance LC-MS/MS and GC-MS/MS instruments, a clear trend towards the development and application of multi-analyte methods in mycotoxin research has been observed [2]. Recent reviews show that the great majority of mycotoxin determination in urine performed in the last year was based on LC-MS/MS [8]. However, a major challenge in urine mycotoxin analysis is the extremely low analyte concentrations present following dietary exposure, in the range of few μg L^−1^. Thus, effective, sensitive, and accurate methods for mycotoxin detection in urine are required. Appropriate sample preparation protocols are crucial to accomplish the desired sensitivity while obtaining acceptable limits of detection (LODs) and quantitation (LOQs). Most of the methods available in the literature are based on traditional extraction techniques such as liquid–liquid extraction (LLE) or solid–liquid extraction (SLE), which have several disadvantages, mainly the high solvent volumes, high amounts of sample, and the long times required for the analysis [9]. In recent years, method simplification and miniaturization was one of the most important trends in sample preparation allowing the use of low sample and solvent volume, fast analysis, and greater efficiency [10,11]. In classical LLE, the addition of an inorganic salt into a miscible mixture forces the formation of a two-phase system leading to selective analytes movement into the organic phase, in a technique known as salting-out liquid–liquid extraction (SALLE) [5]. The well-established QuEChERS (quick, easy, cheap, effective, rugged, and safe) method, based on ACN extraction followed by a salting-out and quick dispersive solid-phase extraction (d-SPE), expands the polarity range of the amenable compounds and allows extract purification by using small amounts of non-chlorinated organic solvents [8]. Dispersive liquid–liquid micro extraction (DLLME), which is based on a ternary component solvent system, enhances the surface area between the organic and the aqueous phase, facilitating the achievement of an equilibrium state, markedly reducing extraction time and enhancing enrichment factors [12,13].

In this study, three extraction methods, namely SALLE, miniQuEChERS, and DLLME, have been evaluated and compared based on analytical parameter data for the quantitative measurement by LC-MS/MS of 11 mycotoxins (aflatoxin B1; AFB1, aflatoxin B2; AFB2, aflatoxin G1; AFG1, aflatoxin G2; AFG2, ochratoxin A; OTA, zearalenone; ZEA, beauvericine; BEA, enniatin A; EN A, enniatin B; EN B, enniatin A1; EN A1 and enniatin B1; EN B1) in human urine.

## 2. Results and Discussion

### 2.1. Method Optimization

Several parameters were evaluated in terms of extraction efficiency for each tested methodology: type and volume of extraction solvent, salt amount, and C18 clean-up. Method optimization was performed by recovery experiments in three replicates using blank urine samples (1 mL) spiked at 20 μg L^−1^ (ENs-BEA), 40 μg L^−1^ (AFs) and 80 μg L^−1^ (OTA and ZEA) for each single compound.

#### 2.1.1. Optimization of SALLE

The following parameters affecting extraction efficiency were evaluated: type and volume of extraction solvent, salt amount, and C18 clean-up step. Single modifications were added to the initial extraction conditions (1 mL of ACN-0.5 g NaCl) to study each single parameter, keeping the non-studied parameters fixed.

**Figure 1:**
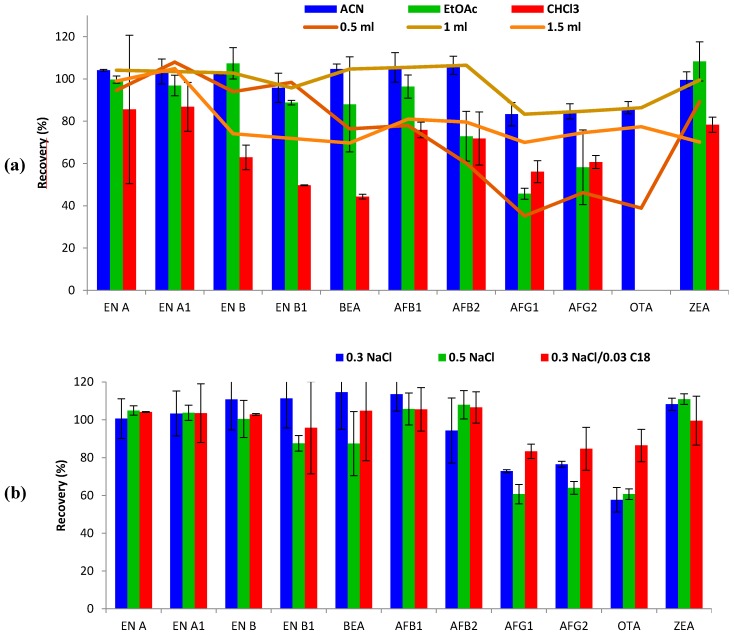
(**a**) Recovery assays performed by salting-out liquid–liquid extraction (SALLE) in urine comparing acetonitrile, ethyl acetate, and chloroform as extraction solvents (vertical bars); acetonitrile volumes of 0.5, 1, 1.5 mL (horizontal lines); and (**b**) different NaCl amounts in the presence/absence of C18 (0.5 g NaCl, 0.3 g NaCl, and 0.3 g NaCl/0.03 g C18, respectively) performed with 1 mL of acetonitrile.

*Type of extraction solvent.* Three different solvents, namely ACN, EtOAc and CHCl_3_, were tested and compared with regard to extraction efficiency for the studied mycotoxins. As shown in Figure 1, the best recovery ranges were obtained with ACN (83–107%), compared to EtOAc (46–108%) and CHCl_3_ (44–87%). For some mycotoxins, such as ENs and ZEA, similar recoveries were obtained with both ACN and EtOAc. However, recoveries of AFs decreased from 83–107% (ACN) to 46–96% when using EtOAc. Moreover, EtOAc and CHCl_3_ did not successfully extract OTA from urine, leading to non-defined chromatographic peaks in terms of symmetry and resolution, therefore reporting inconsistent results. In addition, ACN showed better reproducibility results (RSD < 7%), than EtOAc (RSD < 23%), and CHCl_3_ (RSD < 35%). CHCl_3_ was the least appropriate solvent for mycotoxin extraction in urine using the SALLE method, showing the lowest recovery ranges, even lower than 50% for some mycotoxins (EN B1 and BEA). In keeping with these results, it has been reported that ACN (polarity index: 5.8) is often preferred as the SALLE solvent over other candidates such as acetone, MeOH and ethanol (polarity index: 5.1, 5.1 and 5.2, respectively) [5,14]. Therefore, ACN was selected as the extraction solvent for subsequent optimization testing.

*Volume of extraction solvent.* The effect of the volume of solvent on mycotoxin extraction from 1 mL of urine was evaluated. Volumes of 0.5, 1 and 1.5 mL of ACN were tested (Figure 1). The use of 0.5 mL resulted in an unclear limit between ACN and the saline phase, impeding suitable sampling of the organic phase after centrifugation, thus hindering removal of the upper organic layer. Even so, acceptable recoveries were obtained for some mycotoxins (60–108%, RSD < 11%), but AFGs and OTA did not attain admissible recoveries (<50%). Optimal recovery values were achieved using both 1 mL (83–107%; RSDs < 7%) and 1.5 mL (70–105%; RSDs < 13%) of ACN. Thus, 1 mL of ACN was selected for mycotoxin extraction.

*Sodium chloride amount and C18 clean-up.* SALLE is an alternative sample preparation technique, based on the salting-out effect, to separate water-miscible organic solvents such as ACN. The NaCl amount was first optimized, based on previous studies [5,15,16]. In this way, extraction efficiency was evaluated after adding 0.3 g or 0.5 g of NaCl to urine samples. Since non-significant differences were observed (*p* > 0.05), 0.3 g of NaCl was preferred for miniaturization purposes. Secondly, the influence of the clean-up step was tested by evaluating improvement/worsening after 0.03 g addition of C18 sorbent. The results showed constant recovery results for the great majority of mycotoxins, but an improvement in extraction efficiency (from 58–114% to 83–107%) was observed for AFGs and OTA (Figure 1). Moreover, the addition of C18 led to partial removal of interferences leading to more clean extracts and repeatability improvement (RSD < 7%). Hence, the combination of 0.3 g NaCl followed by 0.03 g C18 clean-up was finally selected for the SALLE procedure performed with ACN.

#### 2.1.2. Optimization of miniQuEChERS

**Figure 2:**
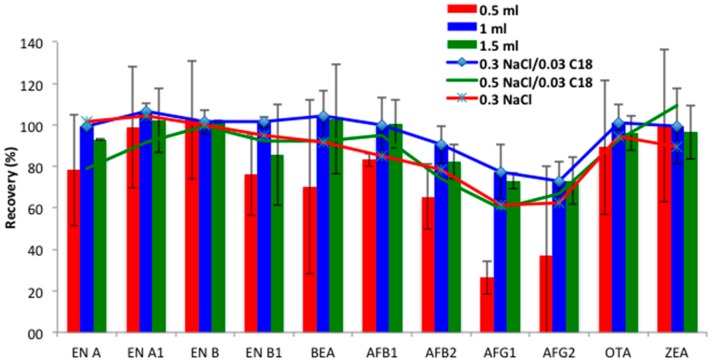
Recovery assays performed by the miniQuEChERS (quick, easy, cheap, effective, rugged, and safe) method in urine comparing acetonitrile volumes of 0.5, 1, 1.5 mL (vertical bars); and using different amounts of MgSO_4_ and C18 (0.5 g NaCl/0.03 g C18; 0.3 g NaCl/0.03 g C18; 0.3 g MgSO_4_, respectively) (horizontal lines).

*Type and volume of extraction solvent.* Since previous studies reported that ACN permitted good recoveries of target mycotoxins and the partial removal of unwanted material from urine, it was proposed as the extraction solvent [17,18]. The extraction efficiency of different ACN volumes (0.5, 1 and 1.5 mL) was evaluated. Similarly to the results obtained in the SALLE procedure, lower volumes (0.5 mL) hampered the organic phase collection, and resulted in lower recovery ranges for some compounds, such as AFs, especially those of group G (25–37%). Moreover, RSDs < 40% revealed that repeatability should be improved for some compounds, such as BEA. Optimal recoveries were achieved using 1 mL (73–107%, RSDs < 18%) and 1.5 mL (73–102%, RSDs < 20%) of ACN (Figure 2). As non-significant differences were found between them (*p* > 0.05), 1 mL of ACN was selected for mycotoxin extraction for the miniQuEChERS procedure.

*Magnesium sulfate amount and C18 clean-up.* QuEChERS is a high effective extraction method, which also allows purification of the extracts [19]. This technique offers different alternatives by re-adjusting protocol according to the analyzed matrix [18]. In the proposed miniQuEChERS methodology, a small volume of 1 mL instead of 10–15 mL as reported for typical QuEChERS method was used. To optimize conditions for the desired purpose, the influence of the MgSO_4_ amount was evaluated by comparing extraction efficiency after addition of 0.3 g and 0.5 g. In this case MgSO_4_ addition was directly combined with the C18 sorbent clean-up step. Thus, the recoveries obtained after ACN extraction with 0.5 g MgSO_4_/0.03 g C18 or 0.3 g MgSO_4_/0.03 g C18 were calculated. As Figure 2 shows, better results were obtained with the lower MgSO_4_ concentration (0.3 g), probably due to its great efficiency in phase separation. MgSO_4_ has more ionic strength (4 mol L^−1^) per unit concentration in aqueous phase than other salts, demonstrating that it is very efficient for phase separation of urine and water-miscible ACN [5]. Finally, in order to check the positive effect of C18 addition, ergo, the clean-up process, the recoveries obtained with 0.3 g NaCl in the absence of C18 were evaluated. As expected, the recovery values decreased for all compounds, except the emerging mycotoxins ENs and BEA, which remained constant (Figure 2). Accordingly, the combination of 0.3 g MgSO_4_ followed by 0.03 g C18 clean-up was selected for the miniQuEChERS procedure.

#### 2.1.3. Optimization of DLLME

The DLLME technique is based on a ternary component solvent system where a disperser solvent and an extraction solvent are combined. The surface area between extraction solvent and aqueous sample is infinitely large; therefore, the equilibrium state is quickly achieved and the extraction time is very short.

*Type of disperser solvent.* First, the type of extraction and disperser solvents was investigated. The miscibility of the disperser solvent with the extraction solvent and aqueous solution is a critical factor in DLLME. With regard to the disperser solvent, it has been reported that when using ACN a cloudy state was correctly formed. Moreover, although other solvents such as acetone, MeOH and ethanol showed suitable properties as disperser solvents, ACN extracts are highly compatible with LC/MS applications resulting in the fewest interferences as large amounts of lipophilic material are not extracted. Hence, based on previous works and other studies reported in the scientific literature [12,14,20,21] 1 mL of ACN was directly selected as the optimum disperser solvent for a 1 mL urine sample size.

In conventional DLLME the density of the extraction solvent was higher than water, therefore its application was limited to water samples and the volume of the sedimented phase was in some cases dependent on the surrounding temperature. Some modification techniques were performed with lower density organic solvents, resulting in improvements in DLLME methods. Thus, DLLME may be classified into two broad categories, depending on the extraction solvent used; lower-density or higher-density solvents [13].

**Figure 3:**
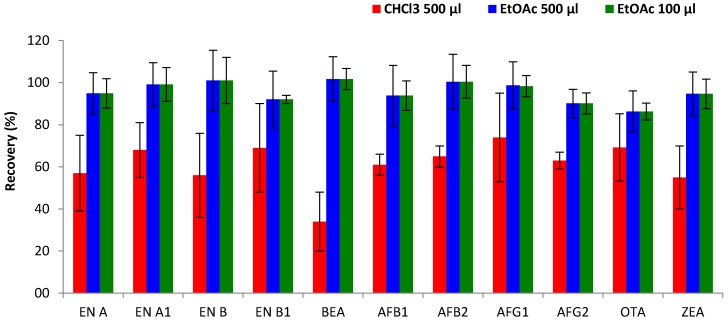
Recovery assays performed by dispersive liquid–liquid microextraction (DLLME) in urine comparing chloroform and ethyl acetate (500 µL), and ethyl acetate (100 µL) as extractant solvents.

*Type of extraction solvent.* The type of extractant solvent is one of the most important parameters that affect the efficiency of DLLME. Therefore, the ability of two extraction solvents to extract the studied mycotoxins from urine was compared: one representative high-density solvent, CHCl_3_ (density 1.49 g/cm^3^); and the other a low-density solvent, EtOAc (density 0.90 g/cm^3^). As shown in Figure 3, better results in terms of extraction efficiency were achieved by EtOAc, showing an improvement from 34–74% (CHCl_3_) to 86–102% (EtOAc) for the great majority of mycotoxins. The highest increase was observed for BEA, which showed low extraction efficiency with CHCl_3_ (34%) but achieved higher recoveries using EtOAc (102%). Only for OTA did the type of extraction solvent show no effect on extraction efficiency and the obtained recoveries (69% for both solvents) remained constant. The better performance of EtOAc compared to CHCl_3_ could be explained by the fact that EtOAc (and other ethers) are hydrogen bond acceptor molecules and therefore extract electron donor solutes more readily than CHCl_3_ [10]. However, some already reported methods achieved good recoveries using CHCl_3_ as the extractant solvent, possibly due to an extraction step being performed before DLLME [21], or the different target compounds [14].

*Volume of extraction solvent.* The effect of the volume of extraction solvent on extraction efficiency was evaluated by different disperser-extraction solvent ratios (1/2 and 1/10), selected on the basis of previous studies [10,21]. Volumes of 500 µL or 100 µL of each extraction solvent (EtOAc, CHCl_3_) were combined with a fixed volume (1 mL) of the disperser solvent (ACN). Results confirmed that better recoveries were obtained with the mixture of ACN containing EtOAc. Moreover, reduction of the extraction volume from 500 µL to 100 µL did not show significant variations in mycotoxin extraction efficiency (Figure 3), with comparable recovery values (86–102% and 70–98%, respectively). On the other hand, a lower volume enhances the enrichment factor of the DLLME process, and reduces the supernatant volume, allowing faster extract evaporation and therefore shortening the extraction time. Thus, 100 µL of EtOAc (extraction solvent) and 1 mL of ACN (disperser solvent) were selected to carry out the DLLME methodology.

*Salt amount influence.* The salting-out effect may significantly improve the analyte extraction in DLLME. It usually increases both the analytes solubility in the organic phase and the drop volume by reducing the solubility of the extractant [16,21]. In this way, the effect of different amounts of added salt (0.1, 0.3 and 0.5 g) on extraction efficiency was tested. In accordance with other authors, it was observed that a small amount of salt (0.1 g) led to an unclear separation phase whereas with larger amounts (0.3 and 0.5 g) the separation between the phases, as well as the peak shape, was better defined [10,22]. Non-significant differences were observed between 0.3 and 0.5 g of NaCl (*p* > 0.05); therefore 0.3 g was selected in order to miniaturize the methodology as much as possible.

### 2.2. Evaluation of the Methods

#### 2.2.1. Salting-Out Liquid–Liquid Extraction (SALLE)

In the SALLE methodology, ACN-NaCl/C18 clean-up was used for mycotoxin extraction. Recoveries ranged between 70 and 108%, with intra-day and inter-day precision lower than 14 and 24%, respectively. Signal enhancement was observed (116–144%) except in the cases of EN B and AFB1, which showed signal suppression (83–85%). Good linearity was observed for all compounds (r^2^ > 0.99). LODs ranged from 0.1 to 10 μg L^−1^, and LOQs were between 0.5 and 40 μg L^−1^ for all mycotoxins (Table 1).

Song et al. [5] compared the SALLE procedure with two fast techniques: (i) dilute-and-shoot; (ii) dilute-evaporate-and-shoot. The authors reported that dilute-evaporate-and-shoot had better sensitivity and response than dilute-and-shoot because of the concentration step (evaporation); however, both techniques showed more serious signal suppression and required a high analyte concentration for a significant signal to be seen. In contrast, the SALLE approach based on two LLE steps with EtOAc and ACN, respectively, gave the highest slope values for all the compounds, which indicated that the matrix effect was minimal. Recoveries ranged from 70 to 108%, with the intra- and inter-day RSD < 25% for most of the compounds and LOQs between 0.07–3.3 μg L^−1^.

Rodriguez-Carrasco et al. [14] selected the SALLE methodology after comparing it with DLLME, for trichothecenes extraction from urine due to its slightly higher extraction efficiency, obtaining recoveries ranging from 84 to 96%, intra-day precision <14%, and LODs/LOQs between 0.12–4 and 0.25–8 μg L^−1^, respectively.

#### 2.2.2. MiniQuEChERS

In the miniQuEChERS methodology, extraction using ACN/MgSO_4_/C18 clean-up was optimized to achieve process simplification in one single step, allowing faster analysis. Table 1 shows the recoveries obtained (71–109%) with intra-day and inter-day precision lower than 14 and 24%, respectively. Signal suppression was observed for the majority of mycotoxins (69–95%) but EN B1, OTA and ZEA showed slight signal enhancement (105–111%). Good linearity was observed for all compounds (r^2^ > 0.99). LODs ranged from 0.1 to 2 μg L^−1^ for all compounds except for ZEA and OTA, which showed the highest values (12 and 15 μg L^−1^, respectively). LOQs ranged between 0.5–6 μg L^−1^ for all mycotoxins except ZEA and OTA (20 and 35 μg L^−1^, respectively).

Rodríguez-Carrasco et al. [15] developed a methodology involving solvent extraction at high ionic strength (ACN/MgSO_4_/NaCl) followed by dispersive solid phase extraction (d-SPE: MgSO_4_/C18) and GC-MS/MS analysis to determine 15 mycotoxins and metabolites in human urine. The methodology was applied by the same authors to evaluate human exposure assessment through mycotoxin/creatinine ratio [23] and to estimate DON excretion through a 24 h pilot study [19]. Similar recoveries to those shown in the present study were achieved (72–109%), with intra and inter-day RSDs < 15%, and LOQs between 0.25 and 8 μg L^−1^. QuEChERS extraction followed by UHPLC-HRMS detection was applied to evaluate mycotoxins and metabolites in human breast milk, showing recoveries ranging from 64% to 93%, RSD < 20%, and lowest calibration levels (LCLs) between 1.25–50 μg L^−1^ [17].

#### 2.2.3. Dispersive Liquid–Liquid Microextraction (DLLME)

DLLME consists of a simple microextraction technique based on the use of an extraction solvent mixed with a disperser solvent. Since DLLME is an efficient, economical, and environmentally responsible methodology, it has gained importance in recent years. It also has other advantages such as simplicity of operation, rapidity, and high recovery and enrichment factors. Nevertheless, there are a limited number of studies that determine mycotoxins using DLLME methodology. Single compounds have been analyzed in different liquid matrices, such as ZEA in beer [12], OTA in wine [24,25], or patulin in apple juice [26]. However, multi-mycotoxin studies applying DLLME methodology are scarce.

In the present study, a DLLME method, based on a low-density extraction solvent (EtOAc) combined with ACN (disperser solvent) in the presence of NaCl, was optimized and successfully validated for the extraction of 11 mycotoxins in urine. As Table 1 shows, satisfactory recoveries were obtained (79–113%) with intra- and inter-day precision lower than 12 and 15%, respectively. Values for the matrix effect ranging from 72 to 117% indicated that both signal enhancement and signal suppression were observed. LODs were between 0.05 and 2 μg L^−1^, and LOQs ranged from 0.5 to 4 μg L^−1^ for all studied mycotoxins. Good linearity was observed for all compounds (r^2^ > 0.99).

Tolosa et al. [21] performed a multi-mycotoxin analysis in water and fish plasma based on a low density-DLLME method using the same solvent mixture ACN/EtOAc (disperser/extraction) with NaCl addition. In line with the results presented here, the authors reported differences in recovery assays when different extraction solvents were employed, achieving better recovery results for the majority of the analyzed mycotoxins when using EtOAc, except for some AFs, which showed better recoveries with CHCl_3_.

Serrano et al. [10] developed a high density DLLME for determining emerging *Fusarium* mycotoxins (ENs and BEA) in water. The extraction was performed in carbon tetrachloride (CCl4, density: 1.59 g/cm^3^) using ACN as the disperser solvent, in the presence of NaCl. Similar recoveries to those obtained in the present study were reported (79 and 100%), with RSD values lower than 14%, and LOQs ranging from 0.06 to 0.17 μg L^−1^.

### 2.3. Selection of the Most Appropriate Methodology

Three extraction methods, namely SALLE, miniQuEChERS and DLLME, were optimized and validated for the extraction of 11 mycotoxins in urine. All the methodologies started from a small sample size (1 mL) in order to follow the current method requirement of simplification and miniaturization. Thus, low sample and solvent volumes were used, with fast analysis and high efficiency. It must be borne in mind that the establishment of feasible multi-biomarker methods is hindered by the absence of the latest generations of QTRAP. Due to the extremely low analyte concentrations present in urine following dietary exposure, the challenge of obtaining acceptable LODs and LOQs must be overcome to achieve the desired sensitivity. The present study therefore focused on parent toxins, to evaluate the efficiency of their extraction through different methodologies. It is expected that the study will be expanded to phase II metabolites in the near future, since it is well known that mycotoxins are widely metabolized in humans and animals. The main analytical parameters for all tested methodologies are compared in Table 1.

*Recovery and matrix effect.* As Table 1 shows, all the studied methodologies showed recoveries within the appropriate range according to the limits set in Commission Decision 2002/657/EC (European Commission 2002) (recoveries: 70–120% and RSDs < 20%). Recoveries for SALLE and miniQuEChERS ranged from 70 to 109%, while DLLME showed recoveries in the range of 73–103%. The matrix effect was evaluated for all the methodologies, showing values from 49% (signal suppression) to 144% (signal enhancement). Since matrix effects represent a major drawback of mycotoxins analysis in complex samples, matrix-matched calibrators were performed to compensate matrix effects and to obtain effective sample quantitation.

*Precision.* Intra-day (repeatability) and inter-day (reproducibility) precision was calculated as the RSD% of triplicate sample measurements (*n* = 3) analyzed on three different non-consecutive days (*n* = 3). Values for intra-day precision were <14% (miniQuEChERS), <14% (SALLE), and <12% (DLLME), indicating better repeatability for the DLLME method. On the other hand, values for inter-day precision were <24% while DLLME showed higher reproducibility (<15%).

Similar recovery and precision results were obtained for trichothecene extraction in urine by SALLE and miniQuEChERS [15,19,21] but higher values of repeatability and reproducibility (<31%) were reported by DLLME [14]. However, DLLME performed for emerging mycotoxins extraction in water and serum samples showed similar precision, as well as recovery values than the obtained in the present study [10,21].

*Sensitivity.* The LODs and LOQs were calculated using the criteria of S/N = 3 and S/N = 10, respectively. Comparable sensitivity results were obtained by the SALLE and miniQuEChERS methods showing LODs between 0.1–10 μg L^−1^ and 0.1–15 μg L^−1^, and LOQs ranging between 0.5–40 μg L^−1^ and 0.5–35 μg L^−1^, respectively. Although satisfactory values were achieved for some mycotoxins, higher LOQs were obtained for OTA and/or ZEA using both methodologies. Sensitivity in multi-mycotoxin methods is hampered by the chemical diversity of analytes, which lead to a necessary compromise between sensitivity, resolution, and analyzed mycotoxins. The lowest LODs and LOQs were achieved by DLLME showing values of a few μg L^−1^ for all the mycotoxins, ranging between 0.05–2 μg L^−1^ and 0.1–4 μg L^−1^, respectively. These values are in concordance with previous studies based on DLLME techniques [10,14,21].

Consequently, DLLME was selected as the most appropriate methodology to extract the studied mycotoxins from urine, achieving high recoveries (73–113%) with RSDs lower than 15% in all cases, and the greatest sensitivity with the lowest reported LOD and LOQ values.

**Figure 4:**
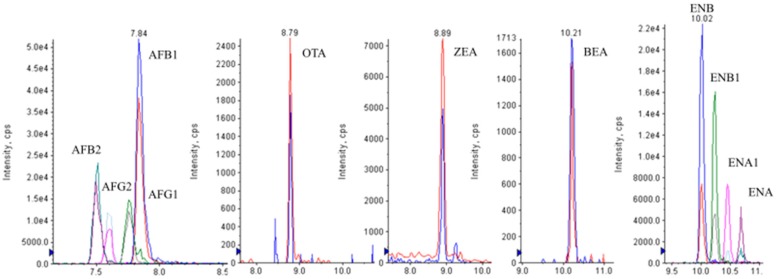
LC-MS/MS chromatograms of a spiked urine sample at 2.5 μg L^−1^ (ENs, BEA), 5 μg L^−1^ (AFs), 20 μg L^−1^ (OTA, ZEA).

Figure 4 shows the LC-MS/MS chromatograms of a spiked urine sample at 2.5 μg L^−1^ (ENs-BEA), 5 μg L^−1^ (AFs), 20 μg L^−1^ (OTA, ZEA).

### 2.4. Human Urine Samples Analysis

The developed method was successfully applied to real urine samples from 10 volunteers of both genders (5 female, 5 male) in an age range of 18–58 years. Urine samples were analyzed in triplicate (*n* = 3) using the developed DLLME methodology. Results showed the presence of ENs B, B1 and A1 in low concentrations. Four samples were positive for EN B (0.1–0.54 μg L^−1^), four detectable but not quantifiable (<LOQ) and two non-detectable (<LOD). With regard to EN B1, two samples reached concentrations of between 0.1 and 0.34 μg L^−1^, while the other four were <LOQs. Finally, EN A was only detected in one sample in concentrations below LOQs. EN A, BEA, AFs, OTA and ZEA were not detected in any urine sample. Two samples showed the simultaneous presence of two mycotoxins in a concentration sum of 0.61 and 0.88 μg L^−1^.

## 3. Conclusions

A new dispersive liquid–liquid microextraction method with a lower-density extraction solvent has been developed for the pre-concentration and quantitative determination of 11 mycotoxins in urine, including AFs, OTA, ZEA, ENs and BEA. Optimized DLLME was selected ahead of miniQuEcHERS and SALLE techniques, as it reported better validation results in terms of recovery, precision and sensitivity. Moreover, the DLLME method has been demonstrated to offer further advantages, such as low operational cost, short extraction time, the use of minimal laboratory material, and environmental friendliness, mainly due to the low solvent volume required. To verify the applicability of the selected methodology, human urine samples from an ongoing pilot survey were analyzed for their mycotoxin levels. Thus, the method was applied to the analysis of real urine samples from healthy volunteers from Valencia, demonstrating its usefulness in human mycotoxin exposure assessment studies, alongside other applications such as toxicokinetics or ADME (absorption, distribution, metabolism, elimination) studies. Further investigations are needed in order to broaden the range of mycotoxins studied and to include their metabolites, conjugates and masked mycotoxins.

## 4. Material and Methods

### 4.1. Chemicals and Reagents

Acetonitrile (ACN), ethyl acetate (EtOAc), methanol (MeOH), chloroform (CHCl_3_) and hexane (HPLC gradient grade, 99.9%) were purchased from Fisher Scientific (Madrid, Spain). Deionized water (<18 MΩ cm^−1^ resistivity) was obtained from a Milli-Q water purification system (Millipore Corp., Bedford, MA, USA). Ammonium formate (HCO_2_NH_4_, 97%) was supplied by Sigma-Aldrich (St. Louis, MO, USA). Chromatographic solvents and water were degassed for 20 min using a Branson 5200 ultrasonic bath (Branson Ultrasonic Corp., Danbury, CT, USA).

### 4.2. Analytical Standards

The standards of AFB1, AFB2, AFG1, AFG2, OTA, ZEA, BEA, EN A, EN B, EN A1 and EN B1 (purity: 99%) were obtained from Sigma-Aldrich. Solid standards were dissolved and combined into a multi-standard working solution for preparation of calibrants and spiking experiments. The standards were stored in darkness and kept at −20 °C until the HPLC-MS/MS analysis.

### 4.3. Sample Collection

Blank urine samples for recovery and validation studies were obtained from five volunteers (age: 25–40) who avoided the consumption of presumably mycotoxin-contaminated food such as cereal-based products for four days. Since no difference (*p* > 0.05) was observed between the blank samples, the validation process was performed by spiking a pooled sample deriving from those five individuals. Additionally, samples from volunteers in Valencia (*n* = 10), including both genders (5 female, 5 male; age 18–58), who were taking part in a larger ongoing human pilot study, were obtained. The Ethics Committee of the University of Valencia approved the project. Informed written consent was obtained from all participants prior to inclusion in the study. The volunteers did not consume any special diet on the days prior to sample donation, but a food questionnaire based on their diet record over four consecutive days was requested in conjunction with urine collection. Participants were screened for their medical history, smoking behavior and pathologies in order to check sampling representativeness. After collection, the samples were stored at −20 °C until analysis.

### 4.4. Samples Extraction Procedures

Several methodologies based on currently employed extraction techniques were tested, optimized, and evaluated in terms of validation data with respect to the main analytical parameters’ linearity, extraction recovery, repeatability, reproducibility, LODs, LOQs and matrix effect, as determined by the European Union (European Commission 2002).

#### 4.4.1. Salting-Out Liquid–Liquid Extraction (SALLE)

One mL of urine was centrifuged at 10,621× *g* for 3 min at 4 °C and the upper layer was placed into a 15 mL test tube. Then, 0.3 g sodium chloride (NaCl), 1 mL of ACN, and 30 mg of C18 sorbent were added in different steps after appropriate mixing. The samples were finally vortexed for 1 min and centrifuged at 4500× *g* for 3 min at 4 °C. The extract was collected as above, filtered and 10 μL were injected into the LC-MS/MS instrument.

#### 4.4.2. MiniQuEChERS

One mL of urine was centrifuged at 10,621× *g* for 3 min at 4 °C and the upper layer was placed into a 15 mL test tube. In this case, 0.3 g magnesium sulfate (MgSO_4_), 1 mL of ACN, and 30 mg of C18 sorbent were added after vigorously mixing between steps. Then, the solution was vortexed again for 1 min and centrifuged at 4500× *g* for 3 min at 4 °C. Finally, the upper layer was collected, filtered and 10 μL were injected into the LC-MS/MS instrument.

#### 4.4.3. Dispersive Liquid–Liquid Microextraction (DLLME)

One mL of centrifuged urine was placed into a 15 mL test tube and 0.3 g sodium chloride was added. After mixing, the mixture of 1 mL of ACN and 100 µL of EtOAc was quickly added. The mixture was vortexed for 1 min and centrifuged at 10,621× *g* for 3 min at 4 °C to achieve the two phases of separation. The supernatant phase was collected, transferred to a vial and evaporated to dryness under a gentle stream of nitrogen. Afterwards, the dry extract was reconstituted with 100 µL of MeOH/water (70:30, *v*/*v*), filtered and 10 μL were injected into the instrument.

### 4.5. HPLC-MS/MS Analysis

HPLC-MS/MS analysis was performed using an Agilent 1200 liquid chromatograph (Agilent Technologies, Palo Alto, CA, USA) coupled to a 3200 QTrap^®^ mass spectrometry system (Applied Biosystems, Foster City, CA, USA) equipped with a Turbo electrospray ionization (ESI) interface. A reversed-phase analytical column (Gemini^®^ C18 column, 3-μm particle size, 150 × 2 mm, I.D.) equipped with a C18 (4 × 2 mm, I.D.; 5 μm security guard cartridge; Phenomenex, Madrid, Spain) was used for analyte chromatographic separation. A binary gradient mode was selected for elution with a constant 0.250 mL/min flow rate. Mobile phases consisted of (A) water/formic acid 99:1 (*v*/*v*) and (B) MeOH/formic acid 99:1 (*v*/*v*), both containing 5 mM ammonium formate. The gradient was programmed as follows: started with 90% A and 10% B (3 min), followed by several linear gradients progressively reaching 70% B (3 min), 80% B (6 min) and 90% B (14 min). The gradient was finally switched back to 90% A (5 min). The injection volume was 10 µL. The QTRAP was used to function as a triple quadrupole mass spectrometry detector (MS/MS) in the multiple selected reaction monitoring (SRM) and using the Turbo V ion spray in positive ionization mode (ESI+). The instrument was operated using the following settings: source temperature 350 °C; ion source gas 1 (sheath gas) 50 psi; ion source gas 2 (drying gas) 55 psi, ion spray voltage 5500 V. The precursor ion of each mycotoxin was confirmed in the product ion scan mode. For each compound, the precursor ion and two characteristic product ions were monitored; using the most abundant for quantitation, and the second one for confirmation. By the acquisition of two SRM transitions per analyte the identification of positive results was confirmed. The criteria applied to confirm mycotoxin identity were: (1) a signal for each of the two SRM transitions of the analyte had to be identical in the sample and in the standard or matrix matched, obtaining four identification points for each analyte; (2) the relative ion intensity of the mycotoxins studied in the standard solution and the spiked samples at the concentration levels used for the calibration curve were compared at tolerance of 0.5% (3) the relative retention time of the analyte in both, sample and standard solution, should be as maximum difference of 0.1 min. Analyst version 1.5.2 software (Applied Biosystem/AB sciex) was used for data acquisition and processing. The final selection of SRM precursor and product transitions, the retention time for each mycotoxin, as well as the optimal declustering potential (DP), collision energies (CE) and collision cell exit potential (CXP) are shown in Table 2.

### 4.6. Method Validation

Method validation followed the guidelines established by the European Union [27]. Validation included the determination of linearity, matrix effect (ME), limits of detection (LODs), limits of quantitation (LOQs), recoveries, repeatability (intra-day precision), and reproducibility (inter-day precision). Calibration curves constructed in standard solutions (external calibrators) and in the matrix (matrix-matched calibrations) were used to evaluate linearity and matrix effects. Matrix-matched calibration curves were prepared by blank samples spiked with selected mycotoxins after extraction. Both external and matrix-matched calibration curves were built by plotting peak areas against concentration and applying linear functions. Eight concentration levels between LOQ and 100 times LOQ were employed to construct the calibration curves, and they were analyzed in triplicate. LODs and LOQs were determined by analysis of decreasing concentrations of the spiked urine, defining them as the concentration with a signal-to-noise ratio (S/N) of 3 and 10, respectively. Matrix effect was assessed by the ratio of (A) the slope of matrix-matched and (B) the slope of external calibration (B), defining matrix effect (%) as follows: A/B × 100. A value of 100% indicated that there was no matrix effect, while a value >100% or <100% represented signal enhancement or signal suppression, respectively. The method’s accuracy was investigated by recovery assays by the repeated analysis of blank urine samples spiked at three concentration levels. For precision evaluation, the relative standard deviation (RSD%) of measurements of three replicates (*n* > 9) was calculated and carried out on the same day (intra-day precision; repeatability), and on three different non-consecutive days (inter-day precision; reproducibility). The spiked levels, selected depending on the method sensitivity, corresponded to 2 LOQs, 5 LOQs and 10 LOQs for low, medium and high levels, respectively. For statistical analysis, a student’s repeated measures *t*-test (*n* = 3) was applied to analyze the results considering as significant *p*-values < 0.05.

## Figures and Tables

**Table 1:** Analytical parameters for *SALLE, miniQuEChERS*, and DLLME method validation: limits of detection (LOD), limits of quantitation (LOQ), recoveries at three spiked concentration levels, intra-day and inter-day precision, matrix effect and linearity for the studied mycotoxins.

Mycotoxin	LOD	LOQ	Recovery (%)			Intra-Day (RSD%)			Inter-Day (RSD%)			Matrix Effect (%)	Linearity (r^2^)
	(μg L^−1^)	(μg L^−1^)	^a^ Spiked Level (μg L^−1^)			^a^ Spiked Level (μg L^−1^)			^a^ Spiked Level (μg L^−1^)				
***SALLE***													
			***low***	***medium***	***high***	***low***	***medium***	***high***	***low***	***medium***	***high***		
**EN A**	1	5	97.8	93.2	86.4	14.1	8.0	6.2	12.0	20.0	19.7	115.6	0.99719
**EN A1**	1	5	104.4	100.5	89.5	11.3	8.4	4.9	17.2	17.8	19.0	122.8	0.99391
**EN B**	0.1	0.5	92.2	95.6	85.6	8.0	8.3	4.5	11.6	16.7	18.8	85.1	0.99944
**EN B1**	0.3	1	88.7	100.7	86.5	6.1	5.0	6.7	7.1	8.7	14.2	124.3	0.99946
**BEA**	3	9	108.4	108.2	89.2	8.6	12.1	8.7	13.5	19.6	19.2	76.3	0.99102
**AFB1**	0.4	1.2	90.0	93.8	82.2	7.6	8.4	6.7	11.5	20.3	19.5	83.3	0.99051
**AFB2**	0.4	1.2	91.3	95.3	85.0	5.2	5.5	6.3	16.2	14.3	19.1	101.5	0.99692
**AFG1**	1.3	4	90.3	91.7	82.3	8.5	7.4	1.5	16.7	15.8	20.0	138.9	0.99519
**AFG2**	1	3	96.7	103.9	84.5	7.7	6.7	5.1	17.4	10.1	15.2	137.7	0.99208
**OTA**	8	20	77.1	72.7	69.7	10.5	8.6	8.1	13.7	18.8	24.4	125.3	0.99360
**ZEA**	10	40	100.4	98.7	87.9	4.9	6.2	3.8	6.3	11.3	14.5	143.8	0.99224
***miniQuEChERS***													
**EN A**	1	5	87.9	89.1	91.3	11.2	4.7	12.4	20.4	11.1	17.0	94.6	0.99615
**EN A1**	0.8	2.5	71.2	106.2	86.9	9.8	8.2	12.9	9.0	15.2	14.5	89.6	0.99473
**EN B**	0.1	0.5	74.9	103.2	91.9	5.4	8.3	4.9	15.2	7.8	9.3	71.2	0.99893
**EN B1**	0.1	0.5	78.2	108.9	79.0	8.7	5.8	9.1	15.2	13.3	24.1	105.1	0.99946
**BEA**	2	6	79.4	100.5	91.8	11.5	6.5	8.6	11.1	8.3	11.3	60.8	0.99715
**AFB1**	0.5	1.5	86.4	94.7	87.4	9.1	5.3	6.0	17.7	20.8	20.0	68.9	0.99804
**AFB2**	0.5	1.5	83.8	98.2	84.5	7.3	6.3	4.3	13.4	19.1	20.4	70.1	0.99854
**AFG1**	1	3	92.3	73.3	76.9	8.7	2.8	8.8	19.3	16.2	22.9	79.0	0.99583
**AFG2**	1	3	85.9	95.8	86.6	8.4	4.3	13.2	15.7	21.1	20.5	87.3	0.99583
**OTA**	15	35	83.8	83.9	74.4	14.4	10.1	4.5	14.7	17.6	15.9	110.3	0.99470
**ZEA**	12	20	99.4	100.7	103.8	5.5	4.5	4.5	11.2	14.1	8.5	110.7	0.97626
***DLLME***													
**EN A**	0.2	0.5	93.7	96.8	94.9	9.9	11.7	7.1	10.2	14.5	10.2	98.3	0.99921
**EN A1**	0.1	0.3	92.6	101.4	99.1	3.9	4.5	6.1	7.0	11.8	10.8	72.0	0.99886
**EN B**	0.05	0.1	99.4	100.7	101.0	11.6	4.3	6.2	8.3	14.6	14.9	99.8	0.99326
**EN B1**	0.05	0.1	93.8	94.9	92.0	9.6	8.7	3.7	9.3	8.1	14.2	79.2	0.99262
**BEA**	0.3	1	93.1	101.2	101.7	5.7	11.6	4.9	14.3	14.6	11.1	70.6	0.99549
**AFB1**	0.1	0.2	94.8	103.5	93.8	7.7	6.1	4.6	12.0	7.5	15.1	116.2	0.99066
**AFB2**	0.2	0.4	94.6	100.7	100.4	3.6	3.0	7.8	6.9	6.5	13.7	109.9	0.99049
**AFG1**	0.2	1	92.3	98.1	98.7	6.0	4.3	5.4	15.4	12.4	11.7	97.2	0.99008
**AFG2**	0.2	0.4	88.0	113.1	90.1	4.7	8.5	5.0	5.7	8.9	7.1	102.6	0.99629
**OTA**	2	4	78.5	82.3	87.2	9.3	9.7	7.6	8.7	10.2	14.5	109.9	0.99792
**ZEA**	1.8	4	93.4	96.9	94.7	4.3	6.5	5.8	7.8	9.7	10.9	109.1	0.99947

^a^ Spiked levels: low (2 LOQ), medium (5 LOQ), high (10 LOQ).

**Table 2:** Optimized MS/MS parameters for the studied mycotoxins: retention time, quantitation transition, confirmation transition, declustering potential (DP), collision energy (CE), and collision cell exit potential (CXP).

Mycotoxin	Retention Time (min)	Quantitation Transition	Confirmation Transition	DP (V)	CE (V)	CXP (V)
**EN A**	13.1	699.4 > 210.1	699.4 > 228.2	76	35	14
**EN A1**	12.2	685.4 > 210.2	685.4 > 214.2	66	37	8
**EN B**	11.1	657.3 > 196.1	657.3 > 214.0	51	39	8
**EN B1**	11.6	671.2 > 214.1	671.2 > 228.1	66	61	10
**BEA**	11.8	801.2 > 784.1	801.2 > 244.1	116	27	10
**AFB1**	8.0	313.1 > 241.0	313.1 > 284.9	46	41	4
**AFB2**	7.9	315.1 > 286.9	315.1 >259.0	81	33	6
**AFG1**	7.8	329.0 > 243.1	329.0 > 311.1	76	39	6
**AFG2**	7.7	331.1 > 313.1	331.1 > 245.1	61	27	6
**OTA**	9.3	404.3 > 102.1	404.3 > 239.0	55	97	6
**ZEA**	9.4	319.0 > 301.0	319.0 > 282.9	26	15	10
